# Moderate to severe food insecurity from pregnancy to 18 months postpartum: a descriptive study, Pelotas, 2016-2020

**DOI:** 10.1590/S2237-96222026v35e20250547.en

**Published:** 2026-01-23

**Authors:** Caroline Nickel Ávila, Carolina Coelho Scholl, Fernanda Teixeira Coelho, Jéssica Puchalski Trettim, Mariana Bonati de Matos, Gabriele Ghisleni, Ana Paula Ardais, Clarissa de Souza Ribeiro Martins, Janaína Vieira dos Santos Motta, Ricardo Tavares Pinheiro, Luciana de Avila Quevedo

**Affiliations:** 1 Universidade Católica de Pelotas, Programa de Pós-Graduação em Saúde e Comportamento, Pelotas, RS, Brazil; 2 Universidade Federal de Pelotas, Programa de Pós-Graduação em Epidemiologia, Pelotas, RS, Brazil

**Keywords:** Food Security, Food Insecurity, Pregnancy, Postpartum Period, Descriptive Studies, Seguridad Alimentaria, Inseguridad Alimentaria, Embarazo, Periodo Posparto, Estudios Descriptivos

## Abstract

**Objective:**

To assess moderate to severe food insecurity and association of its socioeconomic and demographic factors during pregnancy and at 18 months postpartum.

**Methods:**

This was a descriptive study with a representative sample of women up to 24 weeks of pregnancy and at 18 months postpartum. Food insecurity was assessed using the Brazilian Food Insecurity Scale. Households were classified as having moderate to severe food insecurity (no, yes). Absolute and relative frequencies were calculated using the chi-square test.

**Results:**

460 women were assessed. Prevalence of moderate to severe food insecurity was 2.8% during pregnancy, 4.6% at 18 months postpartum, and 3.7% at both points in time. Moderate to severe food insecurity only at 18 months and at both points in time was more frequent in households of women where the head of the household had up to seven years of schooling (10.7% and 9.9%), family income up to R$ 1,500.00 (8.3% and 12.0%), Bolsa Família Program beneficiaries (16.1% and 12.9%), with four or more people living in the household (9.0% and 7.1%), and without paid work (5.0% and 7.2%). It was also higher among those who did not live with a partner (7.8%) and lived in households headed by women (11.0%). Absence of piped water was associated with higher prevalence of moderate to severe food insecurity only during pregnancy (18.2%), only at 18 months (18.2%), and at both times (9.1%).

**Conclusion:**

Moderate to severe food insecurity was related to unfavorable socioeconomic and demographic factors during pregnancy and the postpartum period.

## Resumen

**Objetivo:**

Evaluar la inseguridad alimentaria moderada a grave y su asociación con factores socioeconómicos y demográficos durante el embarazo y a los 18 meses posparto.

**Métodos:**

Estudio descriptivo con una muestra representativa de mujeres hasta las 24 semanas de gestación y a los 18 meses posparto. La inseguridad alimentaria se evaluó mediante la Escala Brasileña de Inseguridad Alimentaria. Los hogares se clasificaron según presentaran inseguridad alimentaria moderada o grave (no, sí). Se calcularon las frecuencias absolutas y relativas mediante la prueba de chi-cuadrado.

**Resultados:**

Se evaluaron 460 mujeres. La prevalencia de inseguridad alimentaria moderada a grave fue del 2,8% durante el embarazo, del 4,6% a los 18 meses posparto y del 3,7% en ambos momentos. La inseguridad alimentaria moderada a grave, tanto a los 18 meses como en ambos momentos, fue más frecuente en hogares de mujeres cuyo jefe de familia tenía hasta siete años de escolaridad (10,7% y 9,9%), ingresos familiares de hasta R$ 1.500,00 (8,3% y 12,0%), beneficiarios del Programa Bolsa Família (16,1% y 12,9%), con cuatro o más personas viviendo en el hogar (9,0% y 7,1%) y sin trabajo remunerado (5,0% y 7,2%). También fue mayor entre quienes no vivían con pareja (7,8%) y residían en hogares encabezados por mujeres (11,0%). La falta de agua potable se asoció con una mayor prevalencia de inseguridad alimentaria moderada a grave únicamente durante el embarazo (18,2%), únicamente a los 18 meses (18,2%) y en ambos momentos (9,1%).

**Conclusión:**

La inseguridad alimentaria moderada a grave se relacionó con factores socioeconómicos y demográficos desfavorables durante el embarazo y el puerperio.

**Ethical aspects**

This research respected ethical principles, having obtained the following approval data:

Research ethics committee: Universidade Católica de Pelotas

Opinion number: Committee approval opinion during pregnancy: 1.174.221 Committee approval opinion at 18 months postpartum: 2.289.620

Approval date: Assessment during pregnancy: approved on 6/8/2015 Assessment at 18 months postpartum: approved on 21/9/2017

Certificate of submission for ethical appraisal: 47807915.4.0000.5339

Informed consent form: All participants signed the consent record.

## Introduction 

Food insecurity is characterized by the lack of regular and permanent access to quality food, in sufficient quantity, without compromising access to other basic needs (1). This condition disproportionately affects vulnerable groups, among which pregnant women and families with young children stand out, being particularly susceptible due to higher nutritional demands and impacts on maternal and child health (2,3). Also at greater risk are individuals living in low-income households, especially those headed by women; Black and Indigenous populations and rural or peripheral communities, whose structural inequalities hinder access to adequate food; as well as people in extreme poverty or homelessness, for whom the absence of support networks exacerbates exposure to food insecurity (4).

Pregnancy is a critical period in the life cycle, characterized by increased energy and nutritional needs to support fetal growth and development, as well as the establishment of energy reserves for lactation (2,3). During this phase, food insecurity can compromise adequate intake of macro and micronutrients through maternal-fetal competition for nutrient availability, which can result in low birth weight, intrauterine growth restriction, premature birth, increased risk of infant mortality, as well as other maternal-fetal complications (2,3,5).

Although food insecurity has been investigated in different population contexts, there are important gaps regarding its prevalence and associated factors during pregnancy and the postpartum period. Pregnant women experiencing food insecurity have a higher risk of adverse outcomes (2,3,5,6), however, specific information on the occurrence of food insecurity throughout the perinatal cycle, including maternal follow-up in the postpartum period, is scarce.

Pregnancy and the postpartum period represent phases of high nutritional vulnerability; therefore, understanding the determinants of moderate to severe food insecurity during this period is fundamental to supporting more effective public policies capable of reducing inequalities and improving maternal and infant outcomes. The objective of this study was to assess moderate to severe food insecurity and association of its socioeconomic and demographic factors during pregnancy and at 18 months postpartum.

## Methods 

### Design

This is a descriptive study that is part of the cohort study entitled “Maternal neuropsychiatric disorders in the pregnancy-puerperal cycle: early detection and intervention and their consequences in the family triad” (7,8), which monitored women from pregnancy until 18 months postpartum.

### Setting

The sampling process occurred in successive stages, with systematic selection of census tracts based on Brazilian Institute of Geography and Statistics data. All 488 tracts of the urban area of ​​Pelotas were listed according to the Brazilian Institute of Geography and Statistics 2010 Census, and 244 were randomly selected for visits by the research team.

From 2016 to 2018, interviewers identified households with women up to 24 weeks pregnant. From 2018 to 2020, participants identified during pregnancy were contacted again and invited for assessment at 18 months postpartum.

### Participants

All women up to 24 weeks pregnant residing in the urban area of ​​Pelotas, a municipality located in the state of Rio Grande do Sul, were invited to participate in the study. The municipality, located in the southernmost part of Brazil, had an estimated population of 328,275 inhabitants, the fourth most populous in the state, with 93% of the population living in the urban area in 2010 (9). The inclusion criterion was self-reported pregnancy. Women with visual impairments or unable to understand and answer the questionnaire were excluded from the research.

### Variables and measurement 

Food insecurity was assessed using the Brazilian Food Insecurity Scale, which consists of 14 items that addressed family experience regarding food sufficiency in the three months prior to the interview (10). Eight items applied to families without children under 18 years of age, and six additional items to those with at least one child under 18 years of age in the household.

The answers were dichotomous (no, yes), and each affirmative response corresponded to one point. The total score defined the scale classification: food security (0 points), mild food insecurity (-5 points for families with children under 18 or 1-3 points for families without children under 18), moderate food insecurity (-10 points for families with children under 18 or 4-6 points for families without children under 18) and severe food insecurity (-14 points for families with children under 18 or 7-8 points for families without children under 18) (10).

Based on the scale levels, a dichotomous variable was created: (i) without moderate to severe food insecurity (food security + mild food insecurity); and (ii) with moderate to severe food insecurity (moderate food insecurity + severe food insecurity). This categorization allowed for four trajectories: without moderate to severe food insecurity during pregnancy and at 18 months postpartum; with moderate to severe food insecurity only during pregnancy; with moderate to severe food insecurity only at 18 months postpartum; and with moderate to severe food insecurity during pregnancy and at 18 months postpartum.

The independent variables, collected at baseline and included in the analysis, were: age range divided into tertiles (≤23, 24-, ≥30 years); living with a partner (no, yes); years of schooling of the head of household (0-, 8-, ≥11 years); paid work (no, yes); monthly family income divided into tertiles (≤R$ 1,500.00; R$ 1,501.00-R$ 2,946.00; ≥R$ 2,947.00); sex of the head of household (female, male, both sexes); presence of piped water in the household (no, yes); receipt of Bolsa Família Program benefit (no, yes); and number of people living in the household (≤2, 3, ≥4).

### Bias

The sample was defined by randomly selecting census tracts from the municipality’s urban area in order to minimize selection bias. This strategy ensured that pregnant women from different socioeconomic and demographic backgrounds had the same probability of participation, which contributed to the representativeness of the sample in relation to the reference population.

### Study size

The sample size calculation considered an estimated food insecurity prevalence rate of 25% in Pelotas, with a 5% margin of error and a 95% confidence level. An additional 30% sample was included to cover possible losses and refusals, resulting in the minimum required size of 372 pregnant women. The sample included in this study exceeded the size calculated to meet the demands of the research to which this investigation belongs.

### Statistical methods

The data were entered into Epidata 3.1 using double entry, with automatic inconsistency checking. Statistical analyses were performed using IBM SPSS software, version 26.0. Absolute and relative frequencies were reported for descriptive analyses. Bivariate analyses were performed using the chi-square test to compare the proportions between the outcome and all independent variables. A 5% significance level and 80% statistical power were assumed for all analyses.

## Results

A total of 1,073 women up to 24 weeks pregnant were identified, of whom 985 were eligible. Two were subsequently excluded due to inability to understand the instruments or lack of confirmation of pregnancy, resulting in a total of 983 participants. At 18 months postpartum, 460 eligible women were located and included in the sample.

Women aged 30 or older represented 35.8% of the sample. The majority lived with a partner (80.9%), and 51.1% lived in households where the head of household had 11 or more years of schooling. More than half of them were employed (56.1%), and 35.1% lived on a monthly family income greater than or equal to R$ 2,947.00.

**Figure 1:**
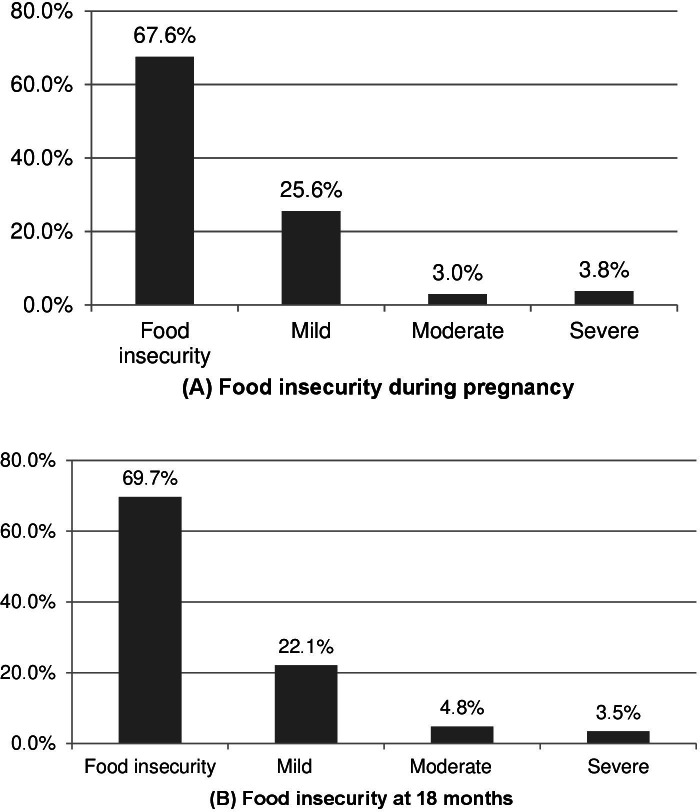
Prevalence (%) of household food insecurity in a sample of women: (A) during pregnancy; (B) at 18 months postpartum. Pelotas, 2016-2020

In 46.9% of households, both men and women shared headship. Most had access to piped water (97.8%), and 9.5% received Bolsa Família Program benefit. Four or more people lived in 37.8% of the households. Prevalence of moderate to severe food insecurity was 2.8% during pregnancy, 4.6% at 18 months postpartum, and 3.7% in both periods (Table 1). Considering the levels of the Brazilian food insecurity scale, prevalence of mild food insecurity during pregnancy was 25.6%; moderate, 3.0%; and severe, 3.8%. At 18 months postpartum, these figures were 22.1%, 4.8% and 3.5%, respectively (Figure 1).

**Table 1:** Absolute (n) and relative (%) frequencies of moderate to severe food insecurity during pregnancy and at 18 months postpartum, according to demographic and socioeconomic variables. Pelotas, 2016-2020 (n=983)

		Moderate to severe food insecurity							
Variables	Total sample	No		During pregnancy		At 18 months postpartum		During pregnancy and at 18 months postpartum	
	n (%)	n (%)	p-value	n (%)	p-value	n (%)	p-value	n (%)	p-value
**Age group** (years) (n=983)			0.476		0.062		0.658		0.613
≤23	323 (32.9)	108 (86.4)		7 (5.6)		4 (3.2)		6 (4.8)	
24-29	308 (31.3)	133 (88.7)		4 (2.7)		7 (4.7)		6 (4.0)	
≥30	352 (35.8)	168 (90.8)		2 (1.1)		10 (5.4)		5 (2.7)	
**Lives with a partner** (n=982)			0.168		0.169		0.365		0.037
No	188 (19.1)	65 (84.4)		4 (5.2)		2 (2.6)		6 (7.8)	
Yes	794 (80.9)	344 (89.8)		9 (2.3)		19 (5.0)		11 (2.9)	
**Head of household years of schooling** (n=958)			<0.001		0.077		<0.001		<0.001
0-7 years	288 (30.1)	89 (73.6)		7 (5.8)		13 (10.7)		12 (9.9)	
8-10 years	180 (18.8)	84 (92.3)		2 (2.2)		2 (2.2)		3 (3.3)	
≥11 years	490 (51.1)	232 (95.9)		4 (1.7)		5 (2.1)		1 (0.4)	
**Paid work** (n=979)			0.007		0.278		0.736		0.001
No	430 (43.9)	152 (84.0)		7 (3.9)		9 (5.0)		13 (7.2)	
Yes	549 (56.1)	257 (92.1)		6 (2.2)		12 (4.3)		4 (1.4)	
**Monthly family income** (BRL) (n=957)			<0.001		0.292		0.010		<0.001
≤1,500.00	320 (33.4)	100 (75.2)		6 (4.5)		11 (8.3)		16 (12.0)	
1,501.00-,946.00	301 (31.5)	134 (92.4)		2 (1.4)		8 (5.5)		1 (0.7)	
≥2,947.00	336 (35.1)	171 (96.1)		5 (2.8)		2 (1.1)		0 (0.0)	
**Head of household’s sex** (n=979)			0.027		0.722		0.605		<0.001
Female	241 (24.6)	75 (82.4)		2 (2.2)		4 (4.4)		10 (11.0)	
Male	279 (28.5)	115 (87.1)		5 (3.8)		8 (6.1)		4 (3.0)	
Both sexes	459 (46.9)	219 (92.4)		6 (2.5)		9 (3.8)		3 (1.3)	
**Household with piped water** (n=979)			<0.001		0.002		0.029		0.337
No	22 (2.2)	6 (54.5)		2 (18.2)		2 (18.2)		1 (9.1)	
Yes	957 (97.8)	403 (89.8)		11 (2.4)		19 (4.2)		16 (3.6)	
**Receives Bolsa Família Program benefit** (n=979)			<0.001		0.207		0.001		0.005
No	886 (90.5)	389 (90.7)		11 (2.6)		16 (3.7)		13 (3.0)	
Yes	93 (9.5)	20 (64.5)		2 (6.5)		5 (16.1)		4 (12.9)	
**Number of people in household** (n=983)			<0.001		0.063		0.003		0.017
≤2	317 (32.2)	153 (95.0)		4 (2.5)		2 (1.2)		2 (1.2)	
3	294 (29.9)	134 (93.1)		1 (0.7)		5 (3.5)		4 (2.8)	
≥4	372 (37.8)	122 (78.7)		8 (5.2)		14 (9.0)		11 (7.1)	
Total	983 (100.0)	409 (88.9)		13 (2.8)		21 (4.6)		17 (3.7)	

Absence of moderate to severe food insecurity during pregnancy and at 18 months postpartum was more prevalent among women living in households where the head of household had 11 or more years of schooling (p-value<0.001), had paid work (p-value 0.007), monthly family income equal to or greater than R$ 2,947.00 (p-value<0.001), and who lived in households with headship shared between men and women (p-value 0.027). This condition was also more frequent among women with access to piped water in their homes (p-value<0.001), who did not receive Bolsa Família Program benefit (p-value<0.001), and who lived in households with up to 2 dwellers (p-value<0.001) (Table 1).

During pregnancy, moderate to severe food insecurity was more prevalent among women without access to piped water (p-value 0.002). At 18 months postpartum, prevalence was higher among those living in households headed by a person with 0-7 years of schooling (p-value<0.001), with a monthly family income of up to R$ 1,500.00 (p-value 0.010), without access to piped water (p-value 0.029), Bolsa Família Program beneficiaries (p-value 0.001), and with 4 or more dwellers (p-value 0.003) (Table 1).

Moderate to severe food insecurity at both points in time investigated, i.e. during pregnancy and at 18 months postpartum, was more prevalent among women without a partner (p-value 0.037), living in households headed by a person with 0-7 years of schooling (p-value<0.001), without paid work (p-value 0.001), with a monthly family income of up to R$ 1,500.00 (p-value<0.001), Bolsa Família Program beneficiaries (p-value 0.005), who lived in households headed by women (p-value<0.001) and with 4 or more dwellers (p-value 0.017) (Table 1).

## Discussion 

This study assessed moderate to severe food insecurity and its association with socioeconomic and demographic factors during pregnancy and at 18 months postpartum. Higher prevalence of moderate to severe food insecurity was found at 18 months postpartum compared to that found during pregnancy or at both periods. Higher prevalence was found among women without access to piped water both during pregnancy and at 18 months postpartum. At 18 months postpartum and at both periods, moderate to severe food insecurity was more prevalent among women living in households that had a head of household with zero to seven years of schooling, with a monthly family income of up to R$ 1,500.00, Bolsa Família Program beneficiaries, and with four or more people living in the same household. In both periods, moderate to severe food insecurity was more frequent among women without a partner, without paid work, and living in households headed by women.

The findings revealed lower prevalence of moderate to severe food insecurity during pregnancy, a period that requires a specific intake of energy and nutrients to meet physiological demands (2,3). Insufficient energy and nutrient intake during this period can lead to maternal-fetal competition for nutrient availability, resulting in adverse outcomes for maternal and infant health (2,3,6).

Higher prevalence of moderate to severe food insecurity at 18 months postpartum may be attributed to the fact that, during this period, all women in this study lived with at least one individual under 18 years of age. Households with individuals under 18 years of age had a higher proportion of food insecurity compared to those without such individuals; the younger the members of the household, the greater the likelihood of experiencing food insecurity (11,12).

Preschool children are a group more susceptible to food insecurity due to their growth and development needs and are disproportionately affected by household food shortages compared to the general population (13). The inability to provide adequate amounts of food for child nutrition can lead to immune and behavioral deficiencies, as well as greater biological vulnerability resulting from micronutrient deficiencies (14).

Experiencing moderate to severe food insecurity both during pregnancy and during 18 months postpartum is particularly concerning; prolonged exposure to this condition during critical developmental phases can lead to unfavorable maternal and child health outcomes (3,6). The severity and duration of exposure to food insecurity can be difficult to quantify due to the variability in the circumstances surrounding this condition (4). The complexity of food insecurity is evidenced by the identification of different trajectories over time. Some children experience persistent food insecurity, while others alternate between mild and moderate levels, or remain consistently food secure. This variation over time demonstrates that individuals can transition between different levels of food insecurity, reflecting the heterogeneous nature of this situation (15).

Socioeconomic and demographic factors significantly influence food insecurity (4). This study identified that households without access to piped water had higher prevalence of moderate to severe food insecurity both during pregnancy and during 18 months postpartum. Poor access to essential basic services can reflect food vulnerability, highlighting the interdependence between food and water insecurity and showing that structural factors, such as family income and access to basic public services, significantly influence food vulnerability (16).

Women with higher prevalence of moderate to severe food insecurity at 18 months postpartum and at both assessment points also lived in households headed by people with low education, lower monthly family income, receiving Bolsa Família Program benefit, and with a larger number of dwellers. Persistent social inequalities contribute to food insecurity and represent important challenges for public health (4).

Low education levels hinder access to the formal labor market, leading to low-paid jobs and potentially contributing to food insecurity (17). When heads of household have low education levels, this also increases the likelihood of a poor-quality diet, due to limited access to information about healthy food choices (18).

Income is one of the main determinants of food insecurity in Brazil (19,20). Access to food depends predominantly on the relationship between income and food prices (19,20). A similar association occurs in the purchasing of poor quality food. Foods with high energy density and low nutritional value tend to be cheaper and more accessible (21).

Families receiving Bolsa Família Program benefit are, for the most part, more vulnerable to food insecurity (22,23). Aimed at families in situations of social vulnerability, the Program aims to ensure food and nutritional security, as well as to reduce social inequalities through government strategies to combat hunger and poverty (22). In this study, the majority of women experiencing moderate to severe food insecurity received Bolsa Família Program benefit, reflecting the socioeconomic vulnerability of its beneficiaries (22,23,24,25).

The study also found higher prevalence of moderate to severe food insecurity in households with a larger number of dwellers. Large families are a common feature in low-income households, and food insecurity can arise as a consequence, since larger families require more food and greater financial resources, which often do not increase proportionally to family size (26).

Women who experienced moderate to severe food insecurity at both points in time were more likely to be unemployed and not living with a partner. Living with a partner can positively influence family socioeconomic status and offer social and emotional support; although it does not guarantee food security, it helps prevent severe food insecurity by providing greater socioemotional and economic stability (17). Absence of paid work is associated with an intergenerational cycle of poverty, characterized by difficulties in entering the formal labor market due to low education, resulting in low income and restricted access to food in adequate quantities and of adequate quality (27).

The head of household plays a crucial role in food insecurity. Households headed by women were associated with moderate to severe food insecurity at both points in time, possibly due to persistent gender disparities in the labor market, such as wage inequality, which implies lower incomes for women. In many cases, women are the sole providers for their families and face a double burden of caregiving and income generation, which can lead to forced unemployment due to domestic and childcare responsibilities (28).

Understanding the findings of this study requires considering the political and economic context experienced in Brazil in recent years. From 2015 onwards, the country faced an economic crisis marked by recession, unemployment and a decline in household income, a situation that worsened with the adoption of fiscal austerity measures after the 2016 parliamentary coup, which reduced the State’s capacity to sustain social policies (29). The dismantling of public social protection policies intensified with the extinction of the National Council for Food and Nutritional Security in January 2019, which weakened participatory governance and intersectoral articulation for the promotion of food security (30). This structural scenario of economic crisis, political instability and institutional dismantling was further aggravated by the COVID-19 pandemic, which intensified poverty and food insecurity, contributing to the persistence of social inequalities (25).

Data collection for this study had to be interrupted in March 2020 due to the COVID-19 pandemic. This resulted in a decrease in the expected sample size for follow-up at 18 months postpartum and was a significant limitation of the study. It is important to emphasize that this is a population-based study that monitored women from pregnancy to child development. Furthermore, the Brazilian food insecurity scale, used to measure household food insecurity, is a direct and validated tool that ensures comparability between studies (10).

The results of this study showed that moderate to severe food insecurity among women during pregnancy and at 18 months postpartum was more prevalent in contexts of greater socioeconomic vulnerability. Although overall prevalence was relatively low, it was found that women in vulnerable situations – such as head of household with a low education level, low monthly family income, participation in the Bolsa Família Program, lack of paid work, large households, female head of household, absence of a partner in the household and lack of access to piped water – presented higher rates of moderate to severe food insecurity, both sporadically and persistently. These findings reinforce the importance of intersectoral public policies aimed at reducing social inequalities and promoting food security, especially among women in situations of greater socioeconomic risk during the pregnancy-puerperium cycle, in order to promote maternal and child quality of life.
