# Supplementary material for: Moderate to severe food insecurity from pregnancy to 18 months postpartum: a descriptive study, Pelotas, 2016-2020
Source: Epidemiol Serv Saude. 2026 Jan 23;35:e20250547. doi: 10.1590/S2237-96222026v35e20250547.en (PMC12829541; doi:10.1590/S2237-96222026v35e20250547.en)
Supplement: Translation

# Translation: Insegurança alimentar moderada a grave da gestação aos 18 meses pós-parto: estudo descritivo, Pelotas, 2016-2020

**DOI:** 10.1590/S2237-96222026v35e20250547.pt

**Authors:** Caroline Nickel Ávila, Carolina Coelho Scholl, Fernanda Teixeira Coelho, Jéssica Puchalski Trettim, Mariana Bonati de Matos, Gabriele Ghisleni, Ana Paula Ardais, Clarissa de Souza Ribeiro Martins, Janaína Vieira dos Santos Motta, Ricardo Tavares Pinheiro, Luciana de Avila Quevedo

**Affiliations:** 1 Universidade Católica de Pelotas, Programa de Pós-Graduação em Saúde e Comportamento, Pelotas, RS, Brasil; 2 Universidade Federal de Pelotas, Programa de Pós-Graduação em Epidemiologia, Pelotas, RS, Brasil

**Keywords:** Segurança Alimentar, Insegurança Alimentar, Gravidez, Período Pós-Parto, Estudos Descritivos

## Abstract

**Objetivo:**

Avaliar a insegurança alimentar moderada a grave e a associação dos seus fatores socioeconômicos e demográficos durante a gestação e aos 18 meses pós-parto.

**Métodos:**

Estudo descritivo com amostra representativa de mulheres com até 24 semanas de gestação e aos 18 meses pós-parto. A insegurança alimentar foi avaliada pela escala brasileira de insegurança alimentar. Os domicílios foram classificados em insegurança alimentar moderada a grave (não, sim). Foram calculadas frequências absolutas e relativas, com a utilização do teste qui-quadrado.

**Resultados:**

Foram avaliadas 460 mulheres. A prevalência de insegurança alimentar moderada a grave foi de 2,8% durante a gestação, de 4,6% aos 18 meses pós-parto e de 3,7% em ambos os momentos. A insegurança alimentar moderada a grave apenas aos 18 meses e em ambos os momentos foi mais frequente em domicílios de mulheres em que o chefe da família tinha até sete anos de escolaridade (10,7% e 9,9%), a renda familiar era de até R$ 1.500,00 (8,3% e 12,0%), beneficiárias do Bolsa Família (16,1% e 12,9%), com quatro ou mais pessoas moradoras (9,0% e 7,1%) e sem trabalho remunerado (5,0% e 7,2%). Também foi maior entre aquelas que não viviam com um companheiro (7,8%) e residiam em domicílios chefiados por mulheres (11,0%). A ausência de água encanada esteve associada à maior prevalência de insegurança alimentar moderada a grave apenas durante a gestação (18,2%), apenas aos 18 meses (18,2%) e em ambos os momentos (9,1%).

**Conclusão:**

A insegurança alimentar moderada a grave esteve relacionada a fatores socioeconômicos e demográficos desfavoráveis no período gestacional e no pós-parto.

**Aspectos éticos**

Esta pesquisa respeitou os princípios éticos, obtendo os seguintes dados de aprovação: :

Comitê de ética em pesquisa: Universidade Católica de Pelotas

Número do parecer: Parecer de aprovação do comitê durante a gestação: 1.174.221 Parecer de aprovação do comitê aos 18 meses pós-parto: 2.289.620

Data de aprovação: Avaliação durante a gestação: aprovado em 6/8/2015 Avaliação aos 18 meses pós-parto: aprovado em 21/9/2017

Certificado de apresentação de apreciação ética: 47807915.4.0000.5339

Registro do consentimento livre e esclarecido: Todas as participantes o assinaram.

## Introdução 

A insegurança alimentar é caracterizada pela falta de acesso regular e permanente a alimentos de qualidade, em quantidade suficiente, sem comprometer o acesso a outras necessidades básicas (1). Essa condição afeta de maneira desproporcional grupos em situação de vulnerabilidade, entre os quais se destacam mulheres grávidas e famílias com crianças pequenas, particularmente suscetíveis devido às maiores demandas nutricionais e aos impactos na saúde materno-infantil (2,3). Também estão em maior risco indivíduos que vivem em domicílios de baixa renda, sobretudo os chefiados por mulheres; populações negras, indígenas e comunidades rurais ou periféricas, cujas desigualdades estruturais dificultam o acesso a alimentos adequados; além de pessoas em situação de pobreza extrema ou sem moradia, para as quais a ausência de redes de apoio agrava a exposição à insegurança alimentar (4).

A gestação é um período crítico do ciclo de vida, caracterizado pelo aumento das necessidades energéticas e nutricionais para sustentar o crescimento e o desenvolvimento fetal, além do estabelecimento de reservas de energia para a lactação (2,3). Nessa fase, a insegurança alimentar pode comprometer a ingestão adequada de macro e de micronutrientes através da competição materno-fetal pela disponibilidade de nutrientes, o que pode resultar em baixo peso ao nascer, restrição do crescimento intrauterino, parto prematuro, maior risco de mortalidade infantil e outras complicações materno-fetais (2,3,5).

Embora a insegurança alimentar já tenha sido investigada em diferentes contextos populacionais, existem lacunas importantes quanto à sua prevalência e aos fatores associados durante a gestação e o período pós-parto. Gestantes em situação de insegurança alimentar apresentam maior risco de desfechos adversos (2,3,5,6), no entanto informações específicas sobre a ocorrência da insegurança alimentar ao longo do ciclo perinatal, com a inclusão do acompanhamento materno no pós-parto, são escassas. 

A gestação e o pós-parto representam fases de elevada vulnerabilidade nutricional, portanto compreender os determinantes da insegurança alimentar moderada a grave nesse período é fundamental para subsidiar políticas públicas mais eficazes, capazes de reduzir desigualdades e melhorar os desfechos materno-infantis. O objetivo deste estudo foi avaliar a insegurança alimentar moderada a grave e a associação dos seus fatores socioeconômicos e demográficos durante a gestação e aos 18 meses pós-parto.

## Métodos 

### Delineamento

Trata-se de estudo descritivo que integra o estudo de coorte “Transtornos neuropsiquiátricos maternos no ciclo gravídico-puerperal: detecção e intervenção precoce e suas consequências na tríade familiar” (7,8), que acompanhou mulheres desde a gestação até 18 meses pós-parto.

### Contexto

O processo de amostragem ocorreu em etapas sucessivas, com seleção sistemática de setores censitários a partir de dados do Instituto Brasileiro de Geografia e Estatística. Todos os 488 setores da área urbana de Pelotas foram listados conforme o Censo de 2010 do Instituto Brasileiro de Geografia e Estatística, e 244 foram sorteados aleatoriamente para visita da equipe de pesquisa. 

De 2016 a 2018, os entrevistadores identificaram domicílios com mulheres de até 24 semanas de gestação. De 2018 a 2020, as participantes identificadas durante a gestação foram novamente contatadas e convidadas para a avaliação aos 18 meses pós-parto. 

### Participantes

Todas as mulheres com até 24 semanas de gestação residentes na área urbana de Pelotas, Rio Grande do Sul, foram convidadas a participar do estudo. O município, localizado no extremo sul do país, tinha população estimada em 328.275 habitantes, o quarto mais populoso do estado, com 93% da população moradora da área urbana em 2010 (9). Como critério de inclusão, considerou-se o autorrelato de gravidez. Mulheres com deficiência visual ou incapazes de entender e responder ao questionário foram excluídas da pesquisa.

### Variáveis e mensuração

A insegurança alimentar foi avaliada pela escala brasileira de insegurança alimentar, composta por 14 itens que abordavam a experiência familiar quanto à suficiência alimentar nos três meses anteriores à entrevista (10). Oito itens aplicaram-se a famílias sem menores de 18 anos, e seis adicionais àquelas com pelo menos um menor de 18 anos no domicílio. 

As respostas foram dicotômicas (não, sim), e cada resposta afirmativa correspondeu a um ponto. O escore total definiu a classificação da escala: segurança alimentar (0 ponto), insegurança alimentar leve (-5 pontos para famílias com menores de 18 anos ou 1-3 pontos para famílias sem esses menores), insegurança alimentar moderada (-10 pontos para famílias com menores de 18 anos ou 4-6 pontos para famílias sem) e insegurança alimentar grave (-14 pontos para famílias com menores de 18 anos ou 7-8 pontos para famílias sem menores de 18 anos) (10). 

Com base nos níveis da escala, foi criada uma variável dicotômica: (i) sem insegurança alimentar moderada a grave (segurança alimentar + insegurança alimentar leve); e (ii) com insegurança alimentar moderada a grave (insegurança alimentar moderada + insegurança alimentar grave). Essa categorização permitiu quatro trajetórias: sem insegurança alimentar moderada a grave durante a gestação e aos 18 meses pós-parto; com insegurança alimentar moderada a grave apenas durante a gestação; com insegurança alimentar moderada a grave apenas aos 18 meses pós-parto; e com insegurança alimentar moderada a grave durante a gestação e aos 18 meses pós-parto.

As variáveis independentes, coletadas na linha de base e incluídas na análise, foram: faixa etária dividida em tercis (≤23, 24-, ≥30 anos); vive com o companheiro (não, sim); tempo de escolaridade do chefe da família (0-, 8-, ≥11 anos); trabalho remunerado (não, sim); renda mensal familiar dividida em tercis (≤R$ 1.500,00; R$ 1.501,00-R$ 2.946,00; ≥R$ 2.947,00); sexo do chefe da família (feminino, masculino, ambos os sexos); presença de água encanada no domicílio (não, sim); recebimento de auxílio do Programa Bolsa Família (não, sim); e número de moradores no domicílio (≤2, 3, ≥4).

### Viés

A amostra foi definida através de sorteio dos setores censitários da zona urbana do município, a fim de minimizar o viés de seleção. Essa estratégia assegurou que gestantes de diferentes contextos socioeconômicos e demográficos tivessem a mesma probabilidade de participação, o que contribuiu para a representatividade da amostra em relação à população de referência.

### Tamanho do estudo

O cálculo amostral considerou a prevalência estimada de 25% de insegurança alimentar em Pelotas, com margem de erro de 5% e nível de confiança de 95%. Uma amostra adicional de 30% foi incluída para cobrir possíveis perdas e recusas, o que resultou no tamanho mínimo necessário de 372 gestantes. A amostra incluída neste estudo superou o tamanho calculado para atender às demandas da pesquisa a qual esta investigação está integrada.

### Métodos estatísticos

Os dados foram digitados no Epidata 3.1 por dupla entrada, com checagem automática de inconsistências. As análises estatísticas foram realizadas no software IBM SPSS, versão 26.0. Foram reportadas as frequências absolutas e relativas para as análises descritivas. Análises bivariadas foram realizadas com a utilização do teste qui-quadrado para comparar as proporções entre o desfecho e todas as variáveis independentes. Assumiram-se nível de significância de 5% e poder de 80% para todas as análises.

## Resultados

Foram identificadas 1.073 mulheres com até 24 semanas de gestação, das quais 985 eram elegíveis. Duas foram posteriormente excluídas por incapacidade de compreender os instrumentos ou por falta de confirmação da gravidez, o que totalizou 983 participantes. Aos 18 meses pós-parto, 460 mulheres elegíveis foram localizadas e incluídas na amostra.

Mulheres com 30 anos ou mais representavam 35,8% da amostra. A maioria vivia com companheiro (80,9%), e 51,1% residiam em domicílios cujo chefe tinha 11 anos ou mais de escolaridade. Mais da metade delas trabalhava de forma remunerada (56,1%) e 35,1% viviam com renda familiar mensal maior ou igual a R$ 2.947,00. 

A chefia era compartilhada entre homens e mulheres em 46,9% dos domicílios. A maioria tinha acesso à água encanada (97,8%) e 9,5% recebiam auxílio do Programa Bolsa Família. Em 37,8% dos domicílios, residiam 4 ou mais pessoas. A prevalência de insegurança alimentar moderada a grave foi 2,8% na gestação, 4,6% aos 18 meses pós-parto e 3,7% em ambos os períodos (Tabela 1). Ao considerar os níveis da escala brasileira de insegurança alimentar, a prevalência de insegurança alimentar leve durante a gestação foi de 25,6%; a moderada, de 3,0%; e a grave, de 3,8%. Aos 18 meses pós-parto, elas corresponderam a 22,1%, 4,8% e 3,5% (Figura 1).

**Tabela 1:** Frequências absolutas (n) e relativas (%) da condição de insegurança alimentar moderada a grave na gestação e aos 18 meses pós-parto, segundo variáveis demográficas e socioeconômicas. Pelotas, 2016-2020 (n=983)

		Insegurança alimentar moderada a grave							
Variáveis	Amostra total	Não		Durante a gestação		Aos 18 meses pós-parto		Durante a gestação e aos 18 meses pós-parto	
	n (%)	n (%)	p-valor	n (%)	p-valor	n (%)	p-valor	n (%)	p-valor
**Faixa etária** (anos) (n=983)			0,476		0,062		0,658		0,613
≤23	323 (32,9)	108 (86,4)		7 (5,6)		4 (3,2)		6 (4,8)	
24-29	308 (31,3)	133 (88,7)		4 (2,7)		7 (4,7)		6 (4,0)	
≥30	352 (35,8)	168 (90,8)		2 (1,1)		10 (5,4)		5 (2,7)	
**Vive com o companheiro** (n=982)			0,168		0,169		0,365		0,037
Não	188 (19,1)	65 (84,4)		4 (5,2)		2 (2,6)		6 (7,8)	
Sim	794 (80,9)	344 (89,8)		9 (2,3)		19 (5,0)		11 (2,9)	
**Tempo de escolaridade do chefe da família** (n=958)			<0,001		0,077		<0,001		<0,001
0-7 anos	288 (30,1)	89 (73,6)		7 (5,8)		13 (10,7)		12 (9,9)	
8-10 anos	180 (18,8)	84 (92,3)		2 (2,2)		2 (2,2)		3 (3,3)	
≥11 anos	490 (51,1)	232 (95,9)		4 (1,7)		5 (2,1)		1 (0,4)	
**Trabalho remunerado** (n=979)			0,007		0,278		0,736		0,001
Não	430 (43,9)	152 (84,0)		7 (3,9)		9 (5,0)		13 (7,2)	
Sim	549 (56,1)	257 (92,1)		6 (2,2)		12 (4,3)		4 (1,4)	
**Renda mensal familiar** (reais) (n=957)			<0,001		0,292		0,010		<0,001
≤1.500,00	320 (33,4)	100 (75,2)		6 (4,5)		11 (8,3)		16 (12,0)	
1.501,00-2.946,00	301 (31,5)	134 (92,4)		2 (1,4)		8 (5,5)		1 (0,7)	
≥2.947,00	336 (35,1)	171 (96,1)		5 (2,8)		2 (1,1)		0 (0,0)	
**Sexo do chefe da família** (n=979)			0,027		0,722		0,605		<0,001
Feminino	241 (24,6)	75 (82,4)		2 (2,2)		4 (4,4)		10 (11,0)	
Masculino	279 (28,5)	115 (87,1)		5 (3,8)		8 (6,1)		4 (3,0)	
Ambos os sexos	459 (46,9)	219 (92,4)		6 (2,5)		9 (3,8)		3 (1,3)	
**Água encanada no domicílio** (n=979)			<0,001		0,002		0,029		0,337
Não	22 (2,2)	6 (54,5)		2 (18,2)		2 (18,2)		1 (9,1)	
Sim	957 (97,8)	403 (89,8)		11 (2,4)		19 (4,2)		16 (3,6)	
**Recebe auxílio do Programa Bolsa Família** (n=979)			<0,001		0,207		0,001		0,005
Não	886 (90,5)	389 (90,7)		11 (2,6)		16 (3,7)		13 (3,0)	
Sim	93 (9,5)	20 (64,5)		2 (6,5)		5 (16,1)		4 (12,9)	
**Número de pessoas no domicílio** (n=983)			<0,001		0,063		0,003		0,017
≤2	317 (32,2)	153 (95,0)		4 (2,5)		2 (1,2)		2 (1,2)	
3	294 (29,9)	134 (93,1)		1 (0,7)		5 (3,5)		4 (2,8)	
≥4	372 (37,8)	122 (78,7)		8 (5,2)		14 (9,0)		11 (7,1)	
Total	983 (100,0)	409 (88,9)		13 (2,8)		21 (4,6)		17 (3,7)	

**Figura 1:** Prevalência (%) de insegurança alimentar domiciliar em uma amostra de mulheres: (A) gestação; (B) aos 18 meses pós-parto. Pelotas, 2016-2020

A ausência de insegurança alimentar moderada a grave na gestação e aos 18 meses pós-parto foi mais prevalente entre mulheres que viviam em domicílios cujo chefe tinha 11 ou mais anos de escolaridade (p-valor<0,001), com trabalho remunerado (p-valor 0,007), renda familiar mensal igual ou superior a R$ 2.947,00 (p-valor<0,001) e que viviam em domicílios com chefia compartilhada entre homens e mulheres (p-valor 0,027). Tal condição também foi mais frequente entre mulheres com acesso à água encanada nos domicílios (p-valor<0,001), que não recebiam auxílio do Programa Bolsa Família (p-valor<0,001) e que residiam em domicílios com até 2 moradores (p-valor<0,001) (Tabela 1).

Durante a gestação, a insegurança alimentar moderada a grave foi mais prevalente entre as mulheres sem acesso à água encanada (p-valor 0,002). Aos 18 meses pós-parto, a prevalência foi maior entre aquelas que residiam em domicílios cujo chefe tinha de 0-7 anos de escolaridade (p-valor<0,001), com renda familiar mensal de até R$ 1.500,00 (p-valor 0,010), sem acesso à água encanada (p-valor 0,029), beneficiários do Programa Bolsa Família (p-valor 0,001) e com 4 ou mais moradores (p-valor 0,003) (Tabela 1).

A insegurança alimentar moderada a grave em ambos os momentos investigados, gestação e 18 meses pós-parto, foi mais prevalente entre as mulheres sem companheiro (p-valor 0,037), residentes em domicílios cujo chefe tinha de 0-7 anos de escolaridade (p-valor<0,001), sem trabalho remunerado (p-valor 0,001), com renda mensal familiar de até R$ 1.500,00 (p-valor<0,001), beneficiárias do Programa Bolsa Família (p-valor 0,005), que viviam em domicílios chefiados por mulheres (p-valor<0,001) e com 4 ou mais moradores (p-valor 0,017) (Tabela 1).

## Discussão 

Este estudo avaliou a insegurança alimentar moderada a grave e a associação dos seus fatores socioeconômicos e demográficos durante a gestação e aos 18 meses pós-parto. Houve maior prevalência de insegurança alimentar moderada a grave aos 18 meses pós-parto em comparação à gestação ou à ocorrência em ambos os momentos. Foi encontrada maior prevalência entre mulheres sem acesso à água encanada tanto na gestação quanto aos 18 meses pós-parto. Aos 18 meses pós-parto e em ambos os momentos, a insegurança alimentar moderada a grave foi mais prevalente entre mulheres que residiam em domicílios cujo chefe tinha de zero a sete anos de escolaridade, com renda familiar mensal de até R$ 1.500,00, beneficiárias do Programa Bolsa Família e com quatro ou mais pessoas morando no mesmo domicílio. Nos dois momentos de forma conjunta, a insegurança alimentar moderada a grave foi mais frequente entre mulheres sem companheiro, sem trabalho remunerado e que residiam em domicílios chefiados por mulheres.

Os achados mostraram menor prevalência de insegurança alimentar moderada a grave durante a gestação, período que requer uma ingestão específica de energia e de nutrientes para atender às demandas fisiológicas (2,3). A ingestão insuficiente de energia e de nutrientes nesse período pode gerar competição materno-fetal pela disponibilidade de nutrientes, o que resulta em desfechos adversos para a saúde materno-infantil (2,3,6).

A maior prevalência de insegurança alimentar moderada a grave aos 18 meses pós-parto pode ser atribuída ao fato de que, nesse período, todas as mulheres deste estudo moravam com pelo menos um indivíduo com menos de 18 anos. Domicílios com indivíduos menores de 18 anos apresentaram maior proporção de insegurança alimentar em comparação àqueles sem tais indivíduos; quanto mais jovens forem os membros do domicílio, maior a probabilidade de vivenciarem insegurança alimentar (11,12). 

As crianças em idade pré-escolar constituem um grupo mais suscetível à insegurança alimentar devido às demandas de crescimento e de desenvolvimento e são desproporcionalmente afetadas pela escassez de alimentos nos domicílios em comparação à população geral (13). A incapacidade de fornecer quantidade adequada de alimentos para a nutrição da criança pode levá-las a deficiências imunológicas e comportamentais, bem como a maior vulnerabilidade biológica decorrente de carências de micronutrientes (14). 

Vivenciar a insegurança alimentar moderada a grave tanto durante a gestação quanto aos 18 meses pós-parto é particularmente preocupante; a exposição prolongada a essa condição em fases críticas do desenvolvimento pode levar a desfechos desfavoráveis para a saúde materno-infantil (3,6). A severidade e o tempo de exposição à insegurança alimentar podem ser difíceis de quantificar, devido à variabilidade nas circunstâncias que envolvem essa condição (4). A complexidade da insegurança alimentar é evidenciada pela identificação de diferentes trajetórias ao longo do tempo. Algumas crianças vivenciam insegurança alimentar persistente, enquanto outras alternam entre níveis leves e moderados, ou permanecem em segurança alimentar consistente. Essa variação ao longo do tempo demonstra que os indivíduos podem transitar entre diferentes níveis de insegurança alimentar, o que reflete a natureza heterogênea dessa situação (15).

Fatores socioeconômicos e demográficos influenciam significativamente a insegurança alimentar (4). Este estudo identificou que domicílios sem acesso à água encanada apresentaram maior prevalência de insegurança alimentar moderada a grave tanto durante a gestação quanto aos 18 meses pós-parto. O acesso precário a serviços básicos essenciais pode refletir a vulnerabilidade alimentar, o que destaca a interdependência entre insegurança alimentar e hídrica e evidencia que fatores estruturais, como renda familiar e acesso a serviços públicos básicos, influenciam significativamente a vulnerabilidade alimentar (16).

Mulheres com maior prevalência de insegurança alimentar moderada a grave aos 18 meses pós-parto e em ambos os momentos avaliados também residiam em domicílios cujo chefe tinha baixa escolaridade, com menor renda familiar mensal, que recebiam auxílio do Programa Bolsa Família e com maior número de moradores. Desigualdades sociais persistentes contribuem para a insegurança alimentar e representam importantes desafios para a saúde pública (4).

A baixa escolaridade dificulta o acesso ao mercado de trabalho formal, o que leva a empregos com baixa remuneração e pode favorecer a insegurança alimentar (17). A baixa escolaridade do chefe da família também aumenta a probabilidade de uma dieta de baixa qualidade, devido ao acesso restrito a informações sobre escolhas alimentares saudáveis (18).

A renda é um dos principais determinantes da insegurança alimentar no Brasil (19,20). O acesso aos alimentos depende predominantemente da relação entre renda e preços deles (19,20). Associação semelhante ocorre na aquisição de alimentos de má qualidade. Alimentos com elevada densidade energética e baixo valor nutricional costumam ser mais baratos e mais acessíveis (21).

As famílias beneficiárias do Programa Bolsa Família são, em sua maioria, mais vulneráveis à insegurança alimentar (22,23). Voltado para famílias em situação de vulnerabilidade social, o programa tem como objetivo assegurar a segurança alimentar e nutricional, além de reduzir as desigualdades sociais por meio de estratégias governamentais de combate à fome e à pobreza (22). Neste estudo, a maioria das mulheres em condição de insegurança alimentar moderada a grave recebia auxílio do Programa Bolsa Família, o que reflete a vulnerabilidade socioeconômica de seus beneficiários (22,23,24,25).

O estudo também encontrou maior prevalência de insegurança alimentar moderada a grave em domicílios com maior número de moradores. Famílias numerosas são uma característica comum em domicílios de baixa renda, e a insegurança alimentar pode surgir como consequência, já que famílias maiores demandam mais alimentos e maiores recursos financeiros, os quais, frequentemente, não aumentam de forma proporcional ao tamanho da família (26).

Mulheres que vivenciaram insegurança alimentar moderada a grave em ambos os momentos foram mais propensas a estarem desempregadas e a não morarem com o companheiro. Viver com o companheiro pode influenciar positivamente o status socioeconômico familiar e oferecer apoio social e emocional; embora não garanta a segurança alimentar, auxilia a prevenir a insegurança alimentar grave ao proporcionar maior estabilidade socioemocional e econômica (17). A ausência de trabalho remunerado está associada a um ciclo intergeracional de pobreza, caracterizado por dificuldades de inserção no mercado de trabalho formal devido à baixa escolaridade, o que resulta em baixa renda e acesso restrito a alimentos em quantidade e qualidade adequadas (27).

A chefia do domicílio desempenha papel crucial na insegurança alimentar. Domicílios chefiados por mulheres estiveram associados à insegurança alimentar moderada a grave em ambos os momentos, possivelmente devido às persistentes disparidades de gênero no mercado de trabalho, como a desigualdade salarial, que implica rendimentos mais baixos para as mulheres. Em muitos casos, as mulheres são as únicas provedoras da família e enfrentam dupla carga, de cuidado e de geração de renda, o que pode levar ao desemprego forçado devido a responsabilidades domésticas e com os filhos (28).

A compreensão dos achados deste estudo requer a consideração do contexto político e econômico vivenciado no Brasil nos últimos anos. A partir de 2015, o país enfrentou uma crise econômica marcada por recessão, desemprego e queda da renda domiciliar, situação que se agravou com a adoção de medidas de austeridade fiscal após o golpe parlamentar de 2016, que reduziu a capacidade do Estado em sustentar políticas sociais (29). O desmonte das políticas públicas de proteção social se intensificou com a extinção do Conselho Nacional de Segurança Alimentar e Nutricional em janeiro de 2019, o que enfraqueceu a governança participativa e a articulação intersetorial para a promoção da segurança alimentar (30). Esse cenário estrutural de crise econômica, instabilidade política e desmonte institucional foi ainda mais agravado pela pandemia de covid-19, que intensificou a pobreza e a insegurança alimentar, o que contribuiu para a persistência das desigualdades sociais (25).

A coleta de dados deste estudo teve de ser interrompida em março de 2020, devido à pandemia de covid-19. Isso resultou na diminuição da amostra esperada do acompanhamento aos 18 meses pós-parto e constituiu uma limitação importante ao estudo. É importante enfatizar que este é um estudo de base populacional, que acompanhou mulheres desde a gestação até o desenvolvimento infantil. Além disso, a escala brasileira de insegurança alimentar, utilizada para mensurar a insegurança alimentar domiciliar, é uma ferramenta direta e validada, que garante a comparabilidade entre estudos (10).

Os resultados deste estudo mostraram que a insegurança alimentar moderada a grave entre mulheres no período gestacional e aos 18 meses pós-parto foi mais prevalente em contextos de maior vulnerabilidade socioeconômica. Embora a prevalência geral tenha sido relativamente baixa, observou-se que mulheres em situações de vulnerabilidade – como baixa escolaridade do chefe da família, baixa renda mensal familiar, participação no Programa Bolsa Família, ausência de trabalho remunerado, domicílios numerosos, chefia domiciliar feminina, ausência de companheiro no domicílio e falta de acesso à água encanada – apresentaram maiores taxas de insegurança alimentar moderada a grave, tanto de forma pontual quanto persistente. Esses achados reforçam a importância de políticas públicas intersetoriais direcionadas à diminuição das desigualdades sociais e à promoção da segurança alimentar, especialmente entre mulheres em situação de maior risco socioeconômico durante o ciclo gravídico-puerperal, o que promove a qualidade de vida materno-infantil.
